# Supplementary material for: Disease coverage of human genome-wide association studies and pharmaceutical research and development
Source: Commun Med (Lond). 2024 Oct 8;4:195. doi: 10.1038/s43856-024-00625-5 (PMC11461613; doi:10.1038/s43856-024-00625-5)
Supplement: Supplementary file 2 — Description of Additional Supplementary Files [file 43856_2024_625_MOESM2_ESM.pdf]

Description of Additional Supplementary Files

File name: Supplementary Data 1

File description - lists all human genes encoding druggable targets and the maximum clinical phase reached by indication.

File name: Supplementary Data 2

File description - includes diseases that are the indication of approved drugs.

File name: Supplementary Data 3

File description - includes diseases that are or have been the indication of drugs in clinical development.

File name: Supplementary Data 4

File description - includes diseases that are or have been indications for drugs in preclinical development.

File name: Supplementary Data 5

File description - includes diseases subjected to GWAS and deposited in the GWAS Catalog or studied by Neale lab.

File name: Supplementary Data 6

File description - shows diseases subjected to GWAS and deposited in the GWAS Catalog or studied by Neale lab and subjected to drug development (preclinical, clinical or approved).

File name: Supplementary Data 7

File description - includes compounds and their targets for drug targets with more than 25 indications with an approved treatment.
